# Inappropriate Use of Proton Pump Inhibitor Among Elderly Patients in British Columbia: What are the Long-term Adverse Events?

**DOI:** 10.2174/1574886318666230726124540

**Published:** 2023-11-10

**Authors:** Mohamed Ben-Eltriki, Manik Chhabra, Alan Cassels, James M. Wright

**Affiliations:** 1 Community Pharmacist, Care First Pharmacy and Wellness Pharmacy Group, Vancouver, British Columbia, Canada;; 2 Therapeutics Initiative, University of British Columbia, Vancouver, British Columbia, Canada;; 3 Department of Pharmacology and Therapeutics, Max Rady College of Medicine, University of Manitoba, Winnipeg, Manitoba, Canada;; 4 Independent Drug Policy Researcher, Victoria, British Columbia, Canada;; 5 Department of Anesthesiology, Pharmacology and Therapeutics & Medicine, Faculty of Medicine, University of British Columbia, Vancouver, British Columbia, Canada

**Keywords:** Proton pump inhibitors, elderly patients, long-term adverse events, chronic kidney disease, dementia, fractures

## Abstract

**Background:**

Proton pump inhibitors (PPIs) are one of the most used classes of drugs. For most indications, PPIs are only recommended up to 8 weeks duration. However, PPI use continues to expand. Regular and prolonged use of PPIs should be avoided because of the risk of adverse events.

**Objectives:**

The main objective of this study was to (1) investigate the extent of PPI usage in people aged 65 or older in the province of British Columbia (BC), Canada, (2) provide an overview of the harms associated with the long-term use of PPIs.

**Methods:**

We examined utilization trends of the PPIs in BC since the year 2009 using PharmaNet, BC’s medication dispensing database where the information is accessible to community pharmacists. We performed a comprehensive literature search for relevant reviews reporting harms associated with long-term use of PPIs. A search was conducted from January 2014 to June 2022.

**Results:**

Between 2000 and 2018 BC’s population grew by 20%, but the use of PPIs escalated to 257%. Of these older British Columbians, 62% had a cumulative exposure exceeding 2 years and 42% exceeded 5 years. This is alarming because the recommended treatment duration is 4-12 weeks for common indications including reflux esophagitis, and duodenal and gastric ulcers. Only 13.5% were dispensed PPIs for 90 days or less. Patients on long-term PPI therapy should be reassessed. Adverse events of PPI use are common among older adults. We identified over 217 systematic reviews published during the last 8 years of specific harms associated with long-term daily usage of PPIs. These harms include increased risks of death, cardiovascular disease, acute renal injury, chronic kidney disease, dementia, fractures, hypomagnesemia, iron deficiency, vitamin B_12_ deficiency, enteric infection (including *C. difficile*), pneumonia, and neoplasia (gastric cancer, carcinoids, and colon cancer), and drug interactions.

**Conclusion:**

This study revealed a high prevalence of PPI use among elderly populations in BC, Canada. The overutilization of PPIs is often a result of failure to re-evaluate the need for continuation of therapy. Published studies identified signals of serious harm from long-term PPI exposure. Healthcare providers with patients can reverse the relentless expansion of long-term PPI exposure by discussing the expected benefits and potential harms.

## 1 INTRODUCTION

Long-term use of PPI is approved by regulators and/or endorsed by gastroenterologists for the prevention of gastric damage associated with adverse effects of other drugs, gastric bleeding, severe esophagitis, or Barrett’s esophagus [1]. PPIs are indicated for long-term use (*i.e*., >8 weeks) in certain conditions such as upper gastrointestinal tract bleeding ulcers; where PPIs are prescribed twice daily for 8-16 weeks, later decreased to once daily [2]. Furthermore, PPI is also used for NSAID prophylaxis or GI bleed prophylaxis if one or more of the following risk factors exists; age >65, history of ulcers, concurrent use of glucocorticoids or anticoagulants/ antiplatelets (for the duration of NSAID therapy) [3]. PPI is prescribed with biopsy-proven Barrett’s esophagus. PPIs are used for other indications such as endoscopic evidence of severe esophagitis (Los Angeles Grade C or D) [4], Gastroesophageal Reflux Disease (>2 x / wk.), GI bleeding from Peptic Ulcer Disease for which a cause (*e.g*., H. pylori or NSAID use) has not been identified or addressed and ongoing hypersecretory conditions [5].

Overuse and misuse of medications in the older population are among the major concerns worldwide. Elderly patients are most prescribed PPIs to prevent age-related heartburn [6]. Epidemiological studies suggest that peptic ulcer disease [7], and Barrett’s esophagus [8] are more common in elderly patients, and PPIs are proven to be beneficial in them [9]. All other patients who are not indicated for long-term use of PPI can be considered for a deprescribing PPIs trial if they are symptom-free and have received appropriate treatment including the duration of therapy [10]. Inappropriate use of PPIs in older adults is more common globally, especially in patients with cognitive impairment, dementia, and other illness [11, 12]. Rababa *et al*. conducted a study among old age nursing home residents with dementia and reported that 92.5% of the study participants were on PPIs for a longer time than recommended by the standard guidelines [11]. However, the literature states evidence on the long-term use of PPIs and the risk of developing dementia in older adults. A significant association between inappropriate PPI use and the risk of dementia and cognitive impairment was reported in a systematic review [13]. Additionally, elderly patients are usually on polypharmacy which predisposes them to develop the risk of developing drug interactions of PPIs with other medications [14-16].

The objective of this study was first to determine the extent of PPI prescribing among elderly patients in the providence of British Columbia (BC) over the past decade. In addition, herein, we present an overview of harms associated with chronic PPI therapy in older adults.

## 2 MATERIALS AND METHODS

### 2.1 PPI Utilization Data Collection

We examined utilization trends of the PPIs in BC between the year 2009 to 2019 using PharmaNet, BC’s medication dispensing database where the information is accessible to community pharmacists. We obtained access to the B.C. Ministry of Health administrative health claims database through a secure access environment. The database contains linkable but de-identified, health service records containing all prescriptions dispensed at community pharmacies, physician services, hospital separations, and vital statistics data in BC.

### 2.2 Literature Review

A search was performed by information specialists from January 2014 to June 2022 in the following databases: PubMed, MEDLINE, EMBASE (through Ovid), the Cochrane Central Register of Controlled Trials (CENTRAL), and the Cochrane Database. The combination of the following medical subheadings (MeSH) and keywords were used for database searching proton pump inhibitors or PPI and adverse events or esomeprazole or pantoprazole or omeprazole or rabeprazole or lansoprazole and any indications. Alternative spellings and abbreviations of the above keywords were also considered with no limitation on the language or the publishing date.

We identified all studies evaluating the potential adverse events of long-term PPI therapy in adults. We included reviews reporting adverse events in adults treated with a PPI for any indication (duration >12 weeks) compared to patients without PPI treatment (no use, placebo, or H2RA use). Two independent investigators assessed study eligibility and synthesized evidence. Data on adverse events were sought, summarized and interpreted herein.

## 3 RESULTS

### 3.1 Long-term Use of PPIs in Older Adults. Is this a Concern?

PPI use in BC increased between 2009 and 2019. BC’s population grew by 20%, but the use of PPIs by 257% [21]. Of these older British Columbians, 62% had a cumulative exposure exceeding 2 years; 42% exceeded 5 years (Table **1**). In contrast, the recommended treatment duration is 4-8 weeks for common indications including reflux esophagitis, and duodenal and gastric ulcers. Only 13.5% were dispensed PPIs for 90 days or less.

### 3.2 What are the Harms Associated with the Long-term Daily usage of PPIs?

Chronic use of PPIs was associated with serious harm that increases with the duration of exposure, age, and comorbidity. From 2749 articles, we identified over 217 systematic reviews published during the last 8 years of specific harms associated with long-term PPIs use. Table **2** shows a summary of the adverse effects reported in the literature. The supplementary file shows a bibliography sorted by harm type.

## 4 DISCUSSION

There appears to be widespread and inappropriate use of PPIs among elderly people in BC, Canada. This study showed that approximately 86.5% of adults in the providence of BC age 65 or above reported using PPI for more than 3 months. This is an alarming reality of the inappropriate use of PPI by the elderly. This review highlights current data regarding potential adverse events of PPIs in the older adult population. The Canadian Association of Gastroenterology states that “don’t maintain long-term PPI therapy for GI symptoms without an attempt to stop/ reduce PPI at least once per year in most patients” [17]. Starting in 2009, Health Canada and other regulators have reported several adverse events associated with PPI use [18]. Emerging evidence suggests long-term use of PPI is associated with adverse outcomes [1]. Further, PPIs are also associated with elevating health care expenditures, in the USA alone PPIs account for 10 billion expenditures annually [19].

### 4.1 What Should be the Role of Healthcare Providers in the Detection of Potentially Inappropriate PPIs Prescriptions?

There is a strong need to cut down on the irrational use of PPIs. Healthcare providers should counsel patients on the long-term benefits and harms based on the results of evidence-based research. As front-line healthcare providers, community pharmacists are easily accessible to elderly patients; they can play an important role in promoting the rational use of PPIs, and they need to be aware of and participate in monitoring adverse events. A non-randomized control study reported that a pharmacist review of indications and the length of PPIs therapy using a PPI intervention form, followed by consulting a physician and a change of prescription of PPIs lead to a 66.1% decrease in PPIs pill count and a 72% cut down in monthly medical expenditure [20].

### 4.2 What are the Challenges Facing Community Pharmacists in Reducing the Inappropriate Use of PPIs?

• **Community pharmacy gets compensated financially for dispensing pills:** Pharmacists need to be involved in stopping a potentially hazardous long-term medicine. The important part of their job, yet it clashes with the business interests of their employer **“Pharmacists should demonstrate professionalism and apply ethical principles in their daily work”.**

• **Heavy workload:** No time for community pharmacists to communicate their findings to the prescribers effectively because of the work pressure.

• **The unwillingness of some physicians** to review and act on documentation sent by pharmacists because they believed they were not adequately reimbursed to do so.

## CONCLUSION

The results of this study show that the prevalence of PPI use is high among older populations. Reducing inappropriate prescribing of PPIs can minimize the potential for adverse events and reduce the cost. Healthcare providers can potentially facilitate shared decisions when discussing PPI therapy with patients and optimize the use of PPI. By synthesizing evidence from published systematic reviews, we hope this study will assist physicians and pharmacists (the key players in averting inappropriate PPI prescription) in counselling patients regarding the risk of adverse events from PPIs.

## Figures and Tables

**Table 1:** Cumulative PPI exposure among patients aged 65 or older who received a PPI in 2019, British Columbia [22].

Cumulative Duration of PPI Exposure (days)*	**Patients N = 225,151** **(22% BC Population ≥ age 65)**	
	**n**	**%**
1 – 90	30,307	13.5
91 -720	56,144	24.9
721-1825	44,325	19.7
> 1825	94,375	41.9

**Note:** *Cumulative exposure was measured over the previous 10 years using PharmaNet data from Jan 1, 2009 to Dec 31, 2019.

**Table 2:** A summary of all adverse events reported in the literature among patients treated with proton pump inhibitors.

Death Hospitalization Cardiovascular events Cerebral ischemic diseases Ischemic cardiac diseases Acute kidney injury Chronic kidney disease Dementia **Cancers** Digestive tract cancers Gastric cancer Colon cancer Colorectal cancer Hepatocellular carcinoma Esophageal Adenocarcinoma Barrett’s Esophagus Pancreatic cancer Bone Fractures / Falls	**Changes in gut microbiome** Gastric fundic gland polyps Small intestinal bacterial overgrowth Gastrointestinal infection Fundic gland polyps Gut dysbiosis Microscopic colitis Clostridium difficile Vitamin B12 deficiency Iron deficiency Anemia Hypomagnesemia Drug interaction **Laboratory findings** Glycemic Control Gastrin Levels and Gastric Histology	Dental implant failure Myopathy Hepatic encephalopathy Pneumonia Childhood asthma Following use in pregnancy Community-acquired pneumonia COPD Tuberculosis COVID-19

## Data Availability

Not applicable.
