# Supplementary material for: Inappropriate Use of Proton Pump Inhibitor Among Elderly Patients in British Columbia: What are the Long-term Adverse Events?
Source: Curr Drug Saf. 2023 Nov 10;19(2):244–7. doi: 10.2174/1574886318666230726124540 (PMC10788903; doi:10.2174/1574886318666230726124540)
Supplement: Supplementary file 1 — Supplementary material is available on the publisher’s website along with the published article. [file CDS-19-244_SD1.pdf]

Supplementary Material

Inappropriate Use of Proton Pump Inhibitor Among Elderly Patients in British Columbia: What are the Long-term Adverse Events?

Mohamed Ben-Eltriki[1,2,3,*], Manik Chhabra[3], Alan Cassels[4] and James M. Wright[2,5]

[1]Community Pharmacist, Care First Pharmacy and Wellness Pharmacy Group, Vancouver, British Columbia, Canada; [2]Therapeutics Initiative, University of British Columbia, Vancouver, British Columbia, Canada; [3]Department of Pharmacology and Therapeutics, Max Rady College of Medicine, University of Manitoba, Winnipeg, Manitoba, Canada; [4]Independent Drug Policy Researcher, Victoria, British Columbia, Canada; [5]Department of Anesthesiology, Pharmacology and Therapeutics & Medicine, Faculty of Medicine, University of British Columbia, Vancouver, British Columbia, Canada

217 Recent Systematic Reviews of Specific Harms
Associated With Proton Pump Inhibitors

Anemia

1. Ali MD. Proton pump inhibitors' use and risk of iron deficiency anaemia: a systematic review and meta-analysis. Curr Rev Clin Exp Pharmacol. 2022 Mar 7.
2. Dirweesh A, Anugwom CM, Li Y, Vaughn BP, Lake J. Proton pump inhibitors reduce phlebotomy burden in patients with HFE-related hemochromatosis: a systematic review and meta-analysis. Eur J Gastroenterol Hepatol. 2021 Oct 1;33(10):1327-1331.

Bone Fractures / Falls

3. Poly TN, Islam MM, Yang HC, Wu CC, Li YJ. Proton pump inhibitors and risk of hip fracture: a meta-analysis of observational studies. Osteoporos Int. 2019;30(1):103-14.
4. Liu J, Li X, Fan L, Yang J, Wang J, Sun J, et al. Proton pump inhibitors therapy and risk of bone diseases: An update meta-analysis. Life Sci. 2019;218:213-23.
5. Lapumnuaypol K, Thongprayoon C, Wijarnpreecha K, Tiu A, Cheungpasitporn W. Risk of Fall in Patients Taking Proton Pump Inhibitors: A Meta-Analysis. QJM : monthly journal of the Association of Physicians. 2019;112(2):115-21.
6. Fan XD, Ayom MIN, Sun WG, Yin PP, Wang XY, Jia A, et al. An updated meta-analysis: The effect of proton pump inhibitor on risk of osteoporosis and fracture. International Journal of Clinical and Experimental Medicine. 2017;10(11):15680-95.
7. Dubcenco E, Beers-Block PM, Kim LP, Schotland P, Levine JG, McCloskey CA, et al. A Proton Pump Inhibitor in the Reformulation Setting: Bioequivalence and Potential Implications for Long-Term Safety. Clinical and Translational Science. 2017;10(5):387-94.
8. Hussain S, Siddiqui AN, Habib A, Hussain MS, Najmi AK. Proton pump inhibitors' use and risk of hip fracture: a systematic review and meta-analysis. Rheumatology international. 2018;38(11):1999-2014.
9. Nassar Y, Richter S. Proton-pump Inhibitor Use and Fracture Risk: An Updated Systematic Review and Meta-analysis. J. 2018;25(3):141-51.
10. Yang SD, Chen Q, Wei HK, Zhang F, Yang DL, Shen Y, et al. Bone fracture and the interaction between bisphosphonates and proton pump inhibitors: a meta-analysis. International journal of clinical and experimental medicine. 2015;8(4):4899-910.
11. Cai D, Feng W, Jiang Q. Acid-suppressive medications and risk of fracture: An updated meta-analysis. International Journal of Clinical and Experimental Medicine. 2015;8(6):8893-904.
12. Aleraij S, Alhowti S, Ferwana M, Abdulmajeed I. Effect of proton pump inhibitors on bone mineral density: A systematic review and meta-analysis of observational studies. Bone Rep. 2020 Nov 10;13:100732.

13. Briganti SI, Naciu AM, Tabacco G, Cesareo R, Napoli N, Trimboli P, Castellana M, Manfrini S, Palermo A. Proton Pump Inhibitors and Fractures in Adults: A Critical Appraisal and Review of the Literature. Int J Endocrinol. 2021 Jan 15;2021:8902367.

14. Chen Jiayun, Li Anan, Lü Zhaohui, Wu Zixuan, Cai Minjie, Huang Xuyan . Effect of long-term use of proton pump inhibitors on bone mineral density and bone metabolism: a Meta-analysis[J]. Chinese Journal of Tissue Engineering Research, 2021, 25(17): 2775-2780.

15. Li J, Xie X, Liu W, Gu F, Zhang K, Su Z, Wen Q, Sui Z, Zhou P, Yu T. Acid-Suppressive Drugs and Risk of Fracture in Children and Young Adults: A Meta-Analysis of Observational Studies. Front Pharmacol. 2021 Aug 6;12:712939.

16. Poly TN, Islam MM, Yang HC, Wu CC, Li YJ. Proton pump inhibitors and risk of hip fracture: a meta-analysis of observational studies. Osteoporos Int. 2019;30(1):103-14.

17. Liu J, Li X, Fan L, Yang J, Wang J, Sun J, et al. Proton pump inhibitors therapy and risk of bone diseases: An update meta-analysis. Life Sci. 2019;218:213-23

18. Lapumnuaypol K, Thongprayoon C, Wijarnpreecha K, Tiu A, Cheungpasitporn W. Risk of Fall in Patients Taking Proton Pump Inhibitors: A Meta-Analysis. QJM : monthly journal of the Association of Physicians. 2019;112(2):115-21.

19. Fan XD, Ayom MIN, Sun WG, Yin PP, Wang XY, Jia A, et al. An updated meta-analysis: The effect of proton pump inhibitor on risk of osteoporosis and fracture. International Journal of Clinical and Experimental Medicine. 2017;10(11):15680-95.

20. Dubcenco E, Beers-Block PM, Kim LP, Schotland P, Levine JG, McCloskey CA, et al. A Proton Pump Inhibitor in the Reformulation Setting: Bioequivalence and Potential Implications for Long-Term Safety. Clinical and Translational Science. 2017;10(5):387-94.

21. Hussain S, Siddiqui AN, Habib A, Hussain MS, Najmi AK. Proton pump inhibitors' use and risk of hip fracture: a systematic review and meta-analysis. Rheumatology international. 2018;38(11):1999-2014.

22. Nassar Y, Richter S. Proton-pump Inhibitor Use and Fracture Risk: An Updated Systematic Review and Meta-analysis. J. 2018;25(3):141-51.

23. Yang SD, Chen Q, Wei HK, Zhang F, Yang DL, Shen Y, et al. Bone fracture and the interaction between bisphosphonates and proton pump inhibitors: a meta-analysis. International journal of clinical and experimental medicine. 2015;8(4):4899-910.

24. Cai D, Feng W, Jiang Q. Acid-suppressive medications and risk of fracture: An updated meta-analysis. International Journal of Clinical and Experimental Medicine. 2015;8(6):8893-904.

25. Aleraij S, Alhowti S, Ferwana M, Abdulmajeed I. Effect of proton pump inhibitors on bone mineral density: A systematic review and meta-analysis of observational studies. Bone Rep. 2020 Nov 10;13:100732.

26. Briganti SI, Naciu AM, Tabacco G, Cesareo R, Napoli N, Trimboli P, Castellana M, Manfrini S, Palermo A. Proton Pump Inhibitors and Fractures in Adults: A Critical Appraisal and Review of the Literature. Int J Endocrinol. 2021 Jan 15;2021:8902367.

27. Chen Jiayun, Li Anan, Lü Zhaohui, Wu Zixuan, Cai Minjie, Huang Xuyan . Effect of long-term use of proton pump inhibitors on bone mineral density and bone metabolism: a Meta-analysis[J]. Chinese Journal of Tissue Engineering Research, 2021, 25(17): 2775-2780.

28. Li J, Xie X, Liu W, Gu F, Zhang K, Su Z, Wen Q, Sui Z, Zhou P, Yu T. Acid-Suppressive Drugs and Risk of Fracture in Children and Young Adults: A Meta-Analysis of Observational Studies. Front Pharmacol. 2021 Aug 6;12:712939.

CANCERS

Gastric Cancer

29. Wan QY, Wu XT, Li N, Du L, Zhou Y. Long-term proton pump inhibitors use and risk of gastric cancer: a meta-analysis of 926 386 participants. Gut. 2018;03:03.

30. Abbas MK, Zaidi ARZ, Robert CA, Thiha S, Malik BH. The Safety of Long-term Daily Usage of a Proton Pump Inhibitor: A Literature Review. Cureus. 2019 Sep 4;11(9):e5563.

31. Cheung KS, Leung WK. Long-term use of proton-pump inhibitors and risk of gastric cancer: a review of the current evidence. Therap Adv Gastroenterol. 2019 Mar 11;12:1756284819834511.

32. Ford AC, Yuan Y, Moayyedi P. *Helicobacter pylori* eradication therapy to prevent gastric cancer: systematic review

and meta-analysis. Gut. 2020 Dec;69(12):2113-2121. doi: 10.1136/gutjnl-2020-320839.

33. Jiang K, Jiang X, Wen Y, Liao L, Liu FB. Relationship between long-term use of proton pump inhibitors and risk of gastric cancer: A systematic analysis. J Gastroenterol Hepatol. 2019 Nov;34(11):1898-1905. doi: 10.1111/jgh.14759.
34. Poly TN, Lin MC, Syed-Abdul S, Huang CW, Yang HC, Li YJ. Proton Pump Inhibitor Use and Risk of Gastric Cancer: Current Evidence from Epidemiological Studies and Critical Appraisal. Cancers (Basel). 2022 Jun 21;14(13):3052.
35. Segna D, Brusselaers N, Glaus D, Krupka N, Misselwitz B. Association between proton-pump inhibitors and the risk of gastric cancer: a systematic review with meta-analysis. Therap Adv Gastroenterol. 2021 Nov 10; 14:17562848211051463.
36. Zeng R, Cheng Y, Luo D, Wang J, Yang J, Jiang L, Zhuo Z, Guo K, Wu H, Leung FW, Sha W, Chen H. Comprehensive analysis of proton pump inhibitors and risk of digestive tract cancers. Eur J Cancer. 2021 Oct;156:190-201.

Hepatocellular carcinoma

37. Zhao J, Hua L, Li N, An R, Liang C. Letter: proton pump inhibitors use and risk of hepatocellular carcinoma: A meta-analysis of observational studies. Alimentary Pharmacology and Therapeutics. 2018;48(8):886-8.
38. Chang TE, Huang YS, Perng CL, Huang YH, Hou MC. Use of proton pump inhibitors and the risk of hepatocellular carcinoma: A systematic review and meta-analysis. J Chin Med Assoc. 2019 Oct;82(10):756-761.
39. Singh A, Hussain S, Jha R, Jayraj AS, Klugar M, Antony B. Proton pump inhibitor use and the risk of hepatocellular carcinoma: A systematic review of pharmacoepidemiological data. J Evid Based Med. 2021 Dec;14(4):278-280.
40. Song HJ, Jiang X, Henry L, Nguyen MH, Park H. Proton pump inhibitors and risk of liver cancer and mortality in patients with chronic liver disease: a systematic review and meta-analysis. Eur J Clin Pharmacol. 2020 Jun;76(6):851-866.

Pancreatic cancer

41. Alkhushaym N, Almutairi AR, Althagafi A, Fallatah SB, Oh M, Martin JR, Babiker HM, McBride A, Abraham I. Exposure to proton pump inhibitors and risk of pancreatic cancer: a meta-analysis. Expert Opin Drug Saf. 2020 Mar;19(3):327-334.
42. Nasser Mubarak Al Khushaym, Abdulaali Almutairi, Saad Fallatah, Abdulhamid Althagafi, Mok Oh, Ali McBride, Hani M. Babiker, and Ivo Abraham. Journal of Clinical Oncology 2019 37:15_suppl, e15755-e15755
43. Hong HE, Kim AS, Kim MR, Ko HJ, Jung MK. Does the Use of Proton Pump Inhibitors Increase the Risk of Pancreatic Cancer? A Systematic Review and Meta-Analysis of Epidemiologic Studies. Cancers (Basel). 2020 Aug 8;12(8):2220.
44. Laoveeravat P, Thavaraputta S, Vutthikraivit W, Suchartlikitwong S, Mingbunjerdsuk T, Motes A, Nugent K, Rakvit A, Islam E, Islam S. Proton pump inhibitors and histamine-2 receptor antagonists on the risk of pancreatic cancer: a systematic review and meta-analysis. QJM. 2020 Feb 1;113(2):100-107.

Colorectal cancer

45. Liu R, Shao Y. The use of a proton pump inhibitor does not increase the risk of colorectal cancer: a meta- analysis. J Gastrointestin Liver Dis. 2020 Dec 13;29(4):686-687.
46. Ma T, Wu M, Jia S, Yang L. Proton pump inhibitors and the risk of colorectal cancer: a systematic review and meta-analysis of observational studies. Int J Colorectal Dis. 2020 Dec;35(12):2157-2169.
47. Patel A, Spychalski P, Antoszewska M, Regula J, Kobiela J. Proton pump inhibitors and colorectal cancer: A systematic review. World J Gastroenterol. 2021 Nov 28;27(44):7716-7733.

Esophageal Adenocarcinoma In Barrett’s Esophagus

48. Hu Q, Sun TT, Hong J, Fang JY, Xiong H, Meltzer SJ. Proton Pump Inhibitors Do Not Reduce the Risk of in Patients with Barrett's Esophagus: A Systematic Review and Meta-Analysis. PLoS ONE. 2017;12(1):e0169691.
49. Chen Y, Sun C, Wu Y, Chen X, Kailas S, Karadsheh Z, Li G, Guo Z, Yang H, Hu L, Zhou Q. Do proton pump inhibitors prevent Barrett's esophagus progression to high-grade dysplasia and esophageal adenocarcinoma? An updated meta-analysis. J Cancer Res Clin Oncol. 2021 Sep;147(9):2681-2691.
50. Li L, Cao Z, Zhang C, Pan W. Risk of esophageal adenocarcinoma in patients with Barrett's esophagus using proton pump inhibitors: a systematic review with meta-analysis and sequential trial analysis. Transl Cancer Res. 2021

Apr;10(4):1620-1627. doi: 10.21037/tcr-20-3362.

51. Yao H, Wang L, Li H, Xu S, Bai Z, Wu Y, Chen H, Goyal H, Qi X. Proton pump inhibitors may reduce the risk of high-grade dysplasia and/or esophageal adenocarcinoma in Barrett's esophagus: a systematic review and meta-analysis. Expert Rev Clin Pharmacol. 2022 Jan;15(1):79-88.

Fundic gland polyps

52. Martin FC, Chenevix-Trench G, Yeomans ND. Systematic review with meta-analysis: fundic gland polyps and proton pump inhibitors. Aliment Pharmacol Ther. 2016;44(9):915-25.

Chronic Liver Disease/ Liver Cirrhosis/ Hepatic Encephalopathy

53. Hwang SJ, Lee DH, Koh SJ, Kim JW, Park HS, Kim BG, Lee KL. Correlation Between Proton Pump Inhibitors and the Complications of Liver Cirrhosis: A Systematic Review and Meta-Analysis. Turk J Gastroenterol. 2022 Jan;33(1):44-52.
54. Wu X, Zhang D, Yu Y, Lou L, Li X. Proton pump inhibitor use and mortality in patients with cirrhosis: a meta-analysis of cohort studies. Biosci Rep. 2020 Jun 26;40(6):BSR20193890. doi: 10.1042/BSR20193890.
55. Ma YJ, Cao ZX, Li Y, Feng SY. Proton pump inhibitor use increases hepatic encephalopathy risk: A systematic review and meta-analysis. World J Gastroenterol. 2019 Jun 7;25(21):2675-2682.
56. Shi D, Zhou Z, Dai Y, Pan X, Cao Q. Proton Pump Inhibitor Therapy and Hepatic Encephalopathy Risk in Cirrhotic Patients: A Systematic Review with Meta-analysis. Clin Drug Investig. 2019 Sep;39(9):847-856. C
57. Bian J, Wang A, Lin J, Wu L, Huang H, Wang S, et al. Association between proton pump inhibitors and hepatic encephalopathy: A meta-analysis. Medicine (Baltimore). 2017;96(17):e6723.
58. Tantai XX, Yang LB, Wei ZC, Xiao CL, Chen LR, Wang JH, Liu N. Association of proton pump inhibitors with risk of hepatic encephalopathy in advanced liver disease: A meta-analysis. World J Gastroenterol. 2019 Jun 7;25(21):2683-2698.
59. Wongtrakul W, Charoenngnam N, Ungprasert P. Use of proton pump inhibitors is associated with a higher risk of pneumonia in cirrhotic patients: a systematic review and meta-analysis. Ann Gastroenterol. 2020 May-Jun;33(3):277-284

Death

60. Ben-Eltriki M, Green CJ, Maclure M, Musini V, Bassett KL, Wright JM. Do proton pump inhibitors increase mortality? A systematic review and in-depth analysis of the evidence. Pharmacol Res Perspect. 2020 Oct;8(5):e00651

Dementia

61. Li M, Luo Z, Yu S, Tang Z. Proton pump inhibitor use and risk of dementia: Systematic review and meta-analysis. Medicine (Baltimore). 2019;98(7):e14422.
62. Batchelor R, Gilmartin JF, Kemp W, Hopper I, Liew D. Dementia, cognitive impairment and proton pump inhibitor therapy: A systematic review. J Gastroenterol Hepatol. 2017;32(8):1426-35.
63. Desai M, Nutalapati V, Srinivasan S, Fathallah J, Dasari C, Chandrasekhar VT, Mohammad B, Kohli D, Vaezi M, Katz PO, Sharma P. Proton pump inhibitors do not increase the risk of dementia: a systematic review and meta-analysis of prospective studies. Dis Esophagus. 2020 Oct 12;33(10):doaa041.
64. Hussain S, Singh A, Zameer S, Jamali MC, Baxi H, Rahman SO, Alam M, Altamish M, Singh AK, Anil D, Hussain MS, Ahmad A, Najmi AK. No association between proton pump inhibitor use and risk of dementia: Evidence from a meta-analysis. J Gastroenterol Hepatol. 2020 Jan;35(1):19-28.
65. Khan MA, Yuan Y, Iqbal U, Kamal S, Khan M, Khan Z, Lee WM, Howden CW. No Association Linking Short-Term Proton Pump Inhibitor Use to Dementia: Systematic Review and Meta-analysis of Observational Studies. Am J Gastroenterol. 2020 May;115(5):671-678.
66. Song YQ, Li Y, Zhang SL, Gao J, Feng SY. Proton pump inhibitor use does not increase dementia and Alzheimer's disease risk: An updated meta-analysis of published studies involving 642305 patients. PLoS One. 2019 Jul 2;14(7):e0219213.
67. Wang H, Tian L, Yan X. No association between acid suppressant use and risk of dementia: an updated meta-analysis. Eur J Clin Pharmacol. 2022 Mar;78(3):375-382. doi: 10.1007/s00228-021-03248-0.
68. Zhang Y, Liang M, Sun C, Song EJ, Cheng C, Shi T, Min M, Sun Y. Proton pump inhibitors use and dementia risk: a meta-analysis of cohort studies. Eur J Clin Pharmacol. 2020 Feb;76(2):139-147.

Diabetes

69. Peng CC, Tu YK, Lee GY, Chang RH, Huang Y, Bukhari K, Tsai YC, Fu Y, Huang HK, Munir KM. Effects of Proton Pump Inhibitors on Glycemic Control and Incident Diabetes: A Systematic Review and Meta-analysis. J Clin Endocrinol Metab. 2021 Oct 21;106(11):3354-3366.

Drug Drug Interactions

70. Shamliyan TA, Middleton M, Borst C. Patient-centered Outcomes with Concomitant Use of Proton Pump Inhibitors and Other Drugs. Clinical Therapeutics. 2017;39(2):404-27.e36.

71. Yucel E, Sancar M, Yucel A, Okuyan B. Adverse drug reactions due to drug-drug interactions with proton pump inhibitors: assessment of systematic reviews with AMSTAR method. Expert Opinion on Drug Safety. 2016;15(2):223-36.

72. Guzman-Prado Y, Vita R, Samson O. Concomitant Use of Levothyroxine and Proton Pump Inhibitors in Patients with Primary Hypothyroidism: a Systematic Review. J Gen Intern Med. 2021 Jun;36(6):1726-1733.

73. Sakuludomkan W, Na Takuathung M, Dukaew N, Koonrungsesomboon N. Drug-Drug Interactions Between Mycophenolic Acid and Proton Pump Inhibitors: A Systematic Review and Meta-Analysis. Ther Drug Monit. 2022 Jun 1;44(3):384-390.

74. Wang X, Song Y, Wang J, He J, Liu R, Li X, Huang H, Zhang J. Effect of proton pump inhibitors on high-dose methotrexate elimination: a systematic review and meta-analysis. Int J Clin Pharm. 2020 Feb;42(1):23-30

Dental implant failure

75. Chappuis V, Avila-Ortiz G, Araujo MG, Monje A. Medication-related dental implant failure: Systematic review and meta-analysis. Clin Oral Implants Res. 2018;29 Suppl 16:55-68.

Hospitalization

76. Wang KN, Bell JS, Chen EYH, Gilmartin-Thomas JFM, Ilomaki J. Medications and Prescribing Patterns as Factors Associated with Hospitalizations from Long-Term Care Facilities: A Systematic Review. Drugs Aging. 2018;35(5):423-57.

INFECTIONS

Bacterial peritonitis

77. Yu T, Tang Y, Jiang L, Zheng Y, Xiong W, Lin L. Proton pump inhibitor therapy and its association with spontaneous bacterial peritonitis incidence and mortality: A meta-analysis. Dig Liver Dis. 2016;48(4):353-9.

78. Zhong HJ, Lin D, Lu ZY, Yang WY, Chen Y. Use of gastric-acid suppressants may be a risk factor for enteric peritonitis in patients undergoing peritoneal dialysis: A meta-analysis. J Clin Pharm Ther. 2018;17:17.

79. Khan MA, Kamal S, Khan S, Lee WM, Howden CW. Systematic review and meta-analysis of the possible association between pharmacological gastric acid suppression and spontaneous bacterial peritonitis. Eur J Gastroenterol Hepatol. 2015;27(11):1327-36.

80. Yu T, Lin L. Proton pump inhibitor therapy and its association with spontaneous bacterial peritonitis incidence and mortality: A meta-analysis. Journal of Digestive Diseases. 2015:95-6.

81. Alhumaid S, Al Mutair A, Al Alawi Z, Zaidi ARZ, Rabaan AA, Elhazmi A, Al-Omari A. Proton pump inhibitors use and risk of developing spontaneous bacterial peritonitis in cirrhotic patients: A systematic review and meta-analysis. Gut Pathog. 2021 Mar 19;13(1):17.

82. Lin L, Hou L, Deng Y, Zhao T, Wang B, Sun C. Acid suppression therapy and its association with spontaneous bacterial peritonitis incidence: A systemic review and meta-analysis. Hepatol Res. 2020 Feb;50(2):233-245.

83. Xu HB, Wang HD, Li CH, Ye S, Dong MS, Xia QJ, Zhang AQ, Pan K, Ge XL, Dong JH. Proton pump inhibitor use and risk of spontaneous bacterial peritonitis in cirrhotic patients: a systematic review and meta-analysis. Genet Mol Res. 2015 Jul 6;14(3):7490-501.

Clostridium Difficile

84. Cao F, Chen CX, Wang M, Liao HR, Wang MX, Hua SZ, et al. Updated meta-analysis of controlled observational studies: proton-pump inhibitors and risk of Clostridium difficile infection. J Hosp Infect. 2018;98(1):4-13.

85. Azab M, Doo L, Doo DH, Elmofti Y, Ahmed M, Cadavona JJ, et al. Comparison of the Hospital-Acquired Clostridium difficile Infection Risk of Using Proton Pump Inhibitors versus Histamine-2 Receptor Antagonists for Prophylaxis and Treatment of Stress Ulcers: A Systematic Review and Meta-Analysis. Gut and liver. 2017;11(6):781-8.

86. Miao-Miao Z, Hui-Lan L, Jia L, Cong L, Zan-Ling Z. Association between use of proton pump inhibitors and the risk of clostridium difficile infection: A meta-analysis. Chinese Journal of Evidence-Based Medicine. 2016;16(3):278-85.
87. Oshima T, Wu L, Li M, Fukui H, Watari J, Miwa H. Magnitude and direction of the association between Clostridium difficile infection and proton pump inhibitors in adults and pediatric patients: a systematic review and meta-analysis. Journal of Gastroenterology. 2018;53(1):84-94.
88. Tariq R, Singh S, Gupta A, Pardi DS, Khanna S. Association of Gastric Acid Suppression With Recurrent Clostridium difficile Infection: A Systematic Review and Meta-analysis. JAMA Internal Medicine. 2017;177(6):784-91.
89. Furuya-Kanamori L, Stone JC, Clark J, McKenzie SJ, Yakob L, Paterson DL, et al. Comorbidities, Exposure to Medications, and the Risk of Community-Acquired Clostridium difficile Infection: a systematic review and meta-analysis. Infect Control Hosp Epidemiol. 2015;36(2):132-41.
90. Deshpande A, Pasupuleti V, Thota P, Pant C, Rolston DD, Hernandez AV, et al. Risk factors for recurrent Clostridium difficile infection: a systematic review and meta-analysis. Infect Control Hosp Epidemiol. 2015;36(4):452-60.
91. Arriola V, Tischendorf J, Musuuza J, Barker A, Rozelle JW, Safdar N. Assessing the Risk of Hospital-Acquired Clostridium Difficile Infection With Proton Pump Inhibitor Use: A Meta-Analysis. Infect Control Hosp Epidemiol. 2016;37(12):1408-17
92. Avendano-Reyes JM. Association between proton pump inhibitors therapy and Clostridium diffcile infection: Myth or fact?. [Spanish]. Medicina Interna de Mexico. 2016;32(5):561-8.
93. D'Silva KM, Mehta R, Mitchell M, Lee TC, Singhal V, Wilson MG, McDonald EG. Proton pump inhibitor use and risk for recurrent Clostridioides difficile infection: a systematic review and meta-analysis. Clin Microbiol Infect. 2021 Jan 16:S1198-743X(21)00035-5.

Community-acquired Enteric Infection

94. Hafiz RA, Wong C, Paynter S, David M, Peeters G. The Risk of Community-Acquired Enteric Infection in Proton Pump Inhibitor Therapy: Systematic Review and Meta-analysis. Annals of Pharmacotherapy. 2018;52(7):613-22.

Community acquired pneumonia

95. Lambert AA, Lam JO, Paik JJ, Ugarte-Gil C, Drummond MB, Crowell TA. Risk of community-acquired pneumonia with outpatient proton-pump inhibitor therapy: a systematic review and meta-analysis. PLoS ONE. 2015;10(6):e0128004.
96. Wang CH, Li CH, Hsieh R, Fan CY, Hsu TC, Chang WC, et al. Proton pump inhibitors therapy and the risk of pneumonia: a systematic review and meta-analysis of randomized controlled trials and observational studies. Expert Opin Drug Saf. 2019;18(3):163-72.
97. Alshamsi F, Belley-Cote E, Cook D, Almenawer SA, Alqahtani Z, Perri D, Thabane L, Al-Omari A, Lewis K, Guyatt G, Alhazzani W. Efficacy and safety of proton pump inhibitors for stress ulcer prophylaxis in critically ill patients: a systematic review and meta-analysis of randomized trials. Crit Care. 2016 May 4;20(1):120.
98. Nguyen PA, Islam M, Galvin CJ, Chang CC, An SY, Yang HC, Huang CW, Li YJ, Iqbal U. Meta-analysis of proton pump inhibitors induced risk of community-acquired pneumonia. Int J Qual Health Care. 2020 Jun 17;32(5):292-299.
99. Tziatzios G, Triantafyllou K, Gkolfakis P. Meta-analysis associates proton pump inhibitor use with higher pneumonia risk in cirrhotic patients: mining for "diamonds" in the coal. Ann Gastroenterol. 2020 Sep-Oct;33(5):544-545.
100. Xun X, Yin Q, Fu Y, He X, Dong Z. Proton Pump Inhibitors and the Risk of Community-Acquired Pneumonia: An Updated Meta-analysis. Ann Pharmacother. 2022 May;56(5):524-532.

Gut Dysbiosis

101. Le Bastard Q, Al-Ghalith GA, Gregoire M, Chapelet G, Javaudin F, Dailly E, et al. Systematic review: human gut dysbiosis induced by non-antibiotic prescription medications. Aliment Pharmacol Ther. 2018;47(3):332-45.
102. Naito Y, Kashiwagi K, Takagi T, Andoh A, Inoue R. Intestinal Dysbiosis Secondary to Proton-Pump Inhibitor Use. Digestion. 2018;97(2):195-204.
103. Li Z, Wu C, Li L, Wang Z, Xie H, He X, et al. Effect of long-term proton pump inhibitor administration on gastric mucosal atrophy: A meta-analysis. Saudi j. 2017;23(4):222-8.

Small intestinal bacterial overgrowth

104. Su T, Lai S, Lee A, He X, Chen S. Meta-analysis: proton pump inhibitors moderately increase the risk of small in-

testinal bacterial overgrowth. Journal of Gastroenterology. 2018;53(1):27-36.

105. Chen B, Kim JJ, Zhang Y, Du L, Dai N. Prevalence and predictors of small intestinal bacterial overgrowth in irritable bowel syndrome: a systematic review and meta-analysis. Journal of Gastroenterology. 2018;14:14.

106. Su T, Lai S, Lee A, He X, Chen S. Meta-analysis: proton pump inhibitors moderately increase the risk of small intestinal bacterial overgrowth. Journal of Gastroenterology. 2018;53(1):27-36.

107. Chen B, Kim JJ, Zhang Y, Du L, Dai N. Prevalence and predictors of small intestinal bacterial overgrowth in irritable bowel syndrome: a systematic review and meta-analysis. Journal of Gastroenterology. 2018;14:14.

Microscopic colitis

108. Zarinafsar S, Matine L, Razzak E, Li K, Kim E, Yang S, et al. Does long-term use of a proton pump inhibitor (PPI) lead to osteopenia or fractures? Journal of Investigative Medicine. 2018;66 (1):168-9.4

Coronavirus Disease

109. Yan C, Chen Y, Sun C, Ahmed MA, Bhan C, Guo Z, Yang H, Zuo Y, Yan Y, Hu L, Sun Y, Li Y, Zhou Q. Does Proton Pump Inhibitor Use Lead to a Higher Risk of Coronavirus Disease 2019 Infection and Progression to Severe Disease? a Meta-analysis. Jpn J Infect Dis. 2022 Jan 24;75(1):10-15.

110. Fatima K, Almas T, Lakhani S, Jahangir A, Ahmed A, Siddiqui A, Rahim A, Qureshi SA, Arshad Z, Golani S, Musheer A. The Use of Proton Pump Inhibitors and COVID-19: A Systematic Review and Meta-Analysis. Trop Med Infect Dis. 2022 Feb 28;7(3):37.

111. Hariyanto TI, Prasetya IB, Kurniawan A. Proton pump inhibitor use is associated with increased risk of severity and mortality from coronavirus disease 2019 (COVID-19) infection. Dig Liver Dis. 2020 Dec;52(12):1410-1412.

112. Li GF, An XX, Yu Y, Jiao LR, Canarutto D, Yu G, Wang G, Wu DN, Xiao Y. Do proton pump inhibitors influence SARS-CoV-2 related outcomes? A meta-analysis. Gut. 2021 Sep;70(9):1806-1808. doi: 10.1136/gutjnl-2020-323366.

113. Pranata R, Huang I, Lawrensia S, Henrina J, Lim MA, Lukito AA, Kuswardhani RAT, Wibawa IDN. Proton pump inhibitor on susceptibility to COVID-19 and its severity: a systematic review and meta-analysis. Pharmacol Rep. 2021 Dec;73(6):1642-1649.

114. Toubasi AA, AbuAnzeh RB, Khraisat BR, Al-Sayegh TN, AlRyalat SA. Proton Pump Inhibitors: Current Use and the Risk of Coronavirus Infectious Disease 2019 Development and its Related Mortality. Meta-analysis. Arch Med Res. 2021 Aug;52(6):656-659.

115. Zippi M, Fiorino S, Budriesi R, Micucci M, Corazza I, Pica R, de Biase D, Gallo CG, Hong W. Paradoxical relationship between proton pump inhibitors and COVID-19: A systematic review and meta-analysis. World J Clin Cases. 2021 Apr 26;9(12):2763-2777.

Tuberculosis

116. Song HJ, Park H, Seo HJ. Association between use of proton pump inhibitors and tuberculosis risk: a systematic review. Int J Tuberc Lung Dis. 2019 Aug 1;23(8):943-951.

Kidney Diseases

117. Sun J, Sun H, Cui M, Sun Z, Li W, Wei J, et al. The use of anti-ulcer agents and the risk of chronic kidney disease: a meta-analysis. Int Urol Nephrol. 2018;50(10):1835-43.

118. Wu B, Shang W, Li Y, Ren Y, Liu Z, Wei H, et al. Association between proton pump inhibitors use and kidney diseases: A meta-analysis. International Journal of Clinical and Experimental Medicine. 2018;11(7):6465-73.

119. Hussain S, Singh A, Habib A, Najmi AK. Proton pump inhibitors use and risk of chronic kidney disease: Evidence-based meta-analysis of observational studies. Clinical Epidemiology and Global Health. 2019;7(1):46-52.

120. Nochaiwong S, Ruengorn C, Awiphan R, Koyratkoson K, Chaisai C, Noppakun K, et al. The association between proton pump inhibitor use and the risk of adverse kidney outcomes: a systematic review and meta-analysis. Nephrology Dialysis Transplantation. 2018;33(2):331-42.

121. Qiu T, Zhou J, Zhang C. Acid-suppressive drugs and risk of kidney disease: A systematic review and meta-analysis. J Gastroenterol Hepatol. 2018;12:12.

122. Hussain S, Singh A, Habib A, Najmi AK. Proton pump inhibitors use and the risk of chronic kidney disease: A systematic review and meta analysis. Nephrology Dialysis Transplantation. 2017:iii553-554.

123. Wu B, Shang W, Li Y, Ren Y, Liu Z, Wei H, et al. Association between proton pump inhibitors use and kidney dis-

eases: A meta-analysis. International Journal of Clinical and Experimental Medicine. 2018;11(7):6465-73.

124. Boonpheng B, Thongprayoon C, Bathini T, Sharma K, Mao MA, Cheungpasitporn W. Proton pump inhibitors and adverse effects in kidney transplant recipients: A meta-analysis. World J Transplant. 2019 Jun 28;9(2):35-47.

125. Cheema E. Investigating the association of proton pump inhibitors with chronic kidney disease and its impact on clinical practice and future research: a review. J Pharm Policy Pract. 2019 Mar 29;12:6. doi: 10.1186/s40545-019-0167-0.

126. Shareef J, Sridhar S, Shariff A. Proton pump inhibitors and the risk of chronic kidney diseases: a critical. Int J Curr Pharm Res. 2020;12(5):11-4.

127. Vengrus CS, Delfino VD, Bignardi PR. Proton pump inhibitors use and risk of chronic kidney disease and end-stage renal disease. Minerva Urol Nephrol. 2021 Aug;73(4):462-470

128. Yang Y, George KC, Shang WF, Zeng R, Ge SW, Xu G. Proton-pump inhibitors use, and risk of acute kidney injury: a meta-analysis of observational studies. Drug Des Devel Ther. 2017 Apr 24;11:1291-1299.

LT use (over 3 months)

129. Islam MM, Poly TN, Walther BA, Dubey NK, Anggraini Ningrum DN, Shabbir SA, et al. Adverse outcomes of long-term use of proton pump inhibitors: a systematic review and meta-analysis. Eur J Gastroenterol Hepatol. 2018;30(12):1395-405.

Pregnancy PPI use

Childhood asthma following use in pregnancy

130. Lai T, Wu M, Liu J, Luo M, He L, Wang X, et al. Acid-Suppressive Drug Use During Pregnancy and the Risk of Childhood Asthma: A Meta-analysis. Pediatrics. 2018;141(2).

Long term in pregnancy

131. Li CM, Zhernakova A, Engstrand L, Wijmenga C, Brusselaers N. Systematic review with meta-analysis: the risks of proton pump inhibitors during pregnancy. Aliment Pharmacol Ther. 2020 Feb;51(4):410-420.

Laboratory findings

Glycemic Control

132. Gomez-Izquierdo JC, Yu OHY. The Influence of Proton-Pump Inhibitors on Glycemic Control: A Systematic Review of the Literature and a Meta-Analysis. Can. 2017;41(4):351-61.

Gastrin Levels and Gastric Histology

133. Lundell L, Vieth M, Gibson F, Nagy P, Kahrilas PJ. Systematic review: the effects of long-term proton pump inhibitor use on serum gastrin levels and gastric histology. Aliment Pharmacol Ther. 2015;42(6):649-63.

Hypomagnesemia

134. Janett S, Camozzi P, Peeters GGAM, Lava SAG, Simonetti GD, Simonetti BG, et al. Hypomagnesemia Induced by Long-Term Treatment with Proton-Pump Inhibitors. Gastroenterology Research and Practice. 2015;2015 (no pagination)(951768).

135. Cheungpasitporn W, Thongprayoon C, Kittanamongkolchai W, Srivali N, Edmonds PJ, Ungprasert P, et al. Proton pump inhibitors linked to hypomagnesemia: a systematic review and meta-analysis of observational studies. Ren Fail. 2015;37(7):1237-41.

136. Chrysant SG. Proton pump inhibitor-induced hypomagnesemia complicated with serious cardiac arrhythmias. Expert Rev Cardiovasc Ther. 2019 May;17(5):345-351.

137. Park CH, Kim EH, Roh YH, Kim HY, Lee SK. The association between the use of proton pump inhibitors and the risk of hypomagnesemia: a systematic review and meta-analysis. PLoS ONE. 2014;9(11):e112558.

138. Srinutta T, Chewcharat A, Takkavatakarn K, Praditpornsilpa K, Eiam-Ong S, Jaber BL, Susantitaphong P. Proton pump inhibitors and hypomagnesemia: A meta-analysis of observational studies. Medicine (Baltimore). 2019 Nov;98(44):e17788.

139. Liao S, Gan L, Mei Z. Does the use of proton pump inhibitors increase the risk of hypomagnesemia: An updated systematic review and meta-analysis. Medicine (Baltimore). 2019 Mar;98(13):e15011.

Myopathy

140. Colmenares EW, Pappas AL. Proton Pump Inhibitors: Risk for Myopathy? Annals of Pharmacotherapy. 2017;51(1):66-71.

Sexual Disorders

141. Ashfaq M, Haroon MZ, Alkahraman YM. Proton pump inhibitors therapy and risk of hyperprolactinemia with associated sexual disorders. Endocr Regul. 2022 Apr 30;56(2):134-147.

Stroke

142. Yang M, He Q, Gao F, Nirantharakumar K, Veenith T, Qin X, Page AT, Wong MCS, Huang J, Kuo ZC, Xia B, Zhang C, He Y, Meng W, Yuan J, Pan Y. Regular use of proton-pump inhibitors and risk of stroke: a population-based cohort study and meta-analysis of randomized-controlled trials. BMC Med. 2021 Dec 3;19(1):316.

AE DATA FROM REVIEWS OF TX COMPARISONS

Tx H Pylori

143. Dong SQ, Singh TP, Wei X, Yao H, Wang HL. Review: A Japanese population-based meta-analysis of vonoprazan versus PPI for Helicobacter pylori eradication therapy: Is superiority an illusion? Helicobacter. 2017;22(6).

144. Nyssen OP, McNicholl AG, Megraud F, Savarino V, Oderda G, Fallone CA, et al. Sequential versus standard triple first-line therapy for Helicobacter pylori eradication. Cochrane Database of Systematic Reviews. 2016(6):CD009034

145. Zeng Y, Ye Y, Liang D, Guo C, Li L. Meta-analysis of the efficacy of lansoprazole and omeprazole for the treatment of H.pylori-associated duodenal ulcer. Int J Physiol Pathophysiol Pharmacol. 2015;7(3):158-64.

146. Wang B, Wang YH, Lv ZF, Xiong HF, Wang H, Yang Y, et al. Review: efficacy and safety of hybrid therapy for Helicobacter pylori infection: a systematic review and meta-analysis. Helicobacter. 2015;20(2):79-88.

147. Ford AC, Gurusamy SK, Delaney B, Forman D, Moayyedi P. Eradication therapy for peptic ulcer disease in Helicobacter pylori-positive people. Cochrane Database of Systematic Reviews. 2016(4).

148. Liu X, Wang H, Lv Z, Wang Y, Wang B, Xie Y, et al. Rescue Therapy with a Proton Pump Inhibitor Plus Amoxicillin and Rifabutin for Helicobacter pylori Infection: A Systematic Review and Meta-Analysis. Gastroenterol Res Pract. 2015;2015:415648.

149. Shinozaki S, Kobayashi Y, Osawa H, Sakamoto H, Hayashi Y, Lefor AK, Yamamoto H. Effectiveness and Safety of Vonoprazan versus Proton Pump Inhibitors for Second-Line Helicobacter pylori Eradication Therapy: Systematic Review and Meta-Analysis. Digestion. 2021;102(3):319-325.

Tx Peptic Ulcer

150. Scally B, Emberson JR, Spata E, Reith C, Davies K, Halls H, et al. Effects of gastroprotectant drugs for the prevention and treatment of peptic ulcer disease and its complications: a meta-analysis of randomised trials. Lancet Gastroenterol Hepatol. 2018;3(4):231-41.

151. Jian Z, Li H, Race NS, Ma T, Jin H, Yin Z. Is the era of intravenous proton pump inhibitors coming to an end in patients with bleeding peptic ulcers? Meta-analysis of the published literature. British Journal of Clinical Pharmacology. 2016;82(3):880-9

152. Gurusamy SK, Pallari E. Medical versus surgical treatment for refractory or recurrent peptic ulcer. Cochrane Database of Systematic Reviews. 2016(3).

153. Kang H, Kim BJ, Choi G, Kim JG. Vonoprazan versus proton pump inhibitors for the management of gastric endoscopic submucosal dissection-induced artificial ulcer: A systematic review with meta-analysis. Medicine (Baltimore). 2019 Jun;98(24):e15860.

Tx GI Bleeding

154. Szabo IL, Matics R, Hegyi P, Garami A, Illes A, Sarlos P, et al. PPIs Prevent Aspirin-Induced Gastrointestinal Bleeding Better than H2RAs. A Systematic Review and Meta-analysis. J. 2017;26(4):395-402.

155. Bang CS, Joo MK, Kim BW, Kim JS, Park CH, Ahn JY, Lee JH, Lee BE, Yang HJ, Cho YK, Park JM, Kim BJ, Jung HK; Korean College of Helicobacter and Upper Gastrointestinal Research. The Role of Acid Suppressants in the Prevention of Anticoagulant-Related Gastrointestinal Bleeding: A Systematic Review and Meta-Analysis. Gut Liver. 2020 Jan 15;14(1):57-66.

156. Wang Y, Ye Z, Ge L, Siemieniuk RAC, Wang X, Wang Y, Hou L, Ma Z, Agoritsas T, Vandvik PO, Perner A,

Møller MH, Guyatt GH, Liu L. Efficacy and safety of gastrointestinal bleeding prophylaxis in critically ill patients: systematic review and network meta-analysis. BMJ. 2020 Jan 6;368:l6744

Tx Upper GI Bleeding After Endoscopy

157. Zhang YS, Li Q, He BS, Liu R, Li ZJ. Proton pump inhibitors therapy vs H2 receptor antagonists therapy for upper gastrointestinal bleeding after endoscopy: A meta-analysis. World Journal of Gastroenterology. 2015;21(20):6341-51.

Tx of GERD

158. Mei J, Yu Y, Ma J, Yu X. Evaluation of the effectiveness of esomeprazole treatment strategies in the management of patients with gastroesophageal reflux disease symptoms: a meta-analysis. Pharmazie. 2016;71(5):285-91.

159. Direct Comparison of the Efficacy and Safety of Vonoprazan Versus Proton-Pump Inhibitors for Gastroesophageal Reflux Disease: A Systematic Review and Meta-Analysis

Tx of GERD in Idiopathic Pulmonary Fibrosis

160. Fidler L, Sitzer N, Shapera S, Shah PS. Treatment of Gastroesophageal Reflux in Patients With Idiopathic Pulmonary Fibrosis: A Systematic Review and Meta-Analysis. Chest. 2018;153(6):1405-15.

Tx Preventing NSAID-associated gastrointestinal toxicity

161. Yuan JQ, Tsoi KK, Yang M, Wang JY, Threapleton DE, Yang ZY, et al. Systematic review with network meta-analysis: comparative effectiveness and safety of strategies for preventing NSAID-associated gastrointestinal toxicity. Aliment Pharmacol Ther. 2016;43(12):1262-75.

Tx of Gastroesophageal Varices

162. Zhu J, Qi X, Yu H, Su C, Guo X. Acid suppression in patients treated with endoscopic therapy for the management of gastroesophageal varices: a systematic review and meta-analysis. Expert rev. 2018;12(6):617-24.

163. Lo EA, Wilby KJ, Ensom MH. Use of proton pump inhibitors in the management of gastroesophageal varices: a systematic review. Annals of Pharmacotherapy. 2015;49(2):207-19.

Tx Chronic Laryngitis

164. Yang Y, Wu H, Zhou J. Efficacy of acid suppression therapy in gastroesophageal reflux disease-related chronic laryngitis. Medicine (Baltimore). 2016;95(40):e4868.

Preventive Tx in Critically Ill Patients / ICU/ Neurocritical care

165. Toews I, George AT, Peter JV, Kirubakaran R, Fontes LES, Ezekiel JPB, et al. Interventions for preventing upper gastrointestinal bleeding in people admitted to intensive care units. Cochrane Database of Systematic Reviews. 2018;6:CD008687.

166. Sridharan K, Sivaramakrishnan G, Gnanaraj J. Pharmacological interventions for stress ulcer prophylaxis in critically ill patients: a mixed treatment comparison network meta-analysis and a recursive cumulative meta-analysis. Expert Opinion on Pharmacotherapy. 2018;19(2):151-8.

167. Barbateskovic M, Marker S, Granholm A, Anthon CT, Krag M, Jakobsen JC, et al. Stress ulcer prophylaxis with proton pump inhibitors or histamin-2 receptor antagonists in adult intensive care patients: a systematic review with meta-analysis and trial sequential analysis. Intensive Care Medicine. 2019;45(2):143-58.

168. Reynolds PM, MacLaren R. Re-evaluating the Utility of Stress Ulcer Prophylaxis in the Critically Ill Patient: A Clinical Scenario-Based Meta-Analysis. Pharmacotherapy. 2018;13:13.

169. Sridharan K, Sivaramakrishnan G, Gnanaraj J. Pharmacological interventions for stress ulcer prophylaxis in critically ill patients: a mixed treatment comparison network meta-analysis and a recursive cumulative meta-analysis. Expert Opinion on Pharmacotherapy. 2018;19(2):151-8.

170. Toews I, George AT, Peter JV, Kirubakaran R, Fontes LES, Ezekiel JPB, et al. Interventions for preventing upper gastrointestinal bleeding in people admitted to intensive care units. Cochrane Database of Systematic Reviews. 2018;6:CD008687.

171. Liu B, Liu S, Yin A, Siddiqi J. Risks and benefits of stress ulcer prophylaxis in adult neurocritical care patients: a systematic review and meta-analysis of randomized controlled trials. Crit Care. 2015;19:409.

172. Alshamsi F, Belley-Cote E, Cook D, Almenawer SA, Alqahtani Z, Perri D, et al. Efficacy and safety of proton pump inhibitors for stress ulcer prophylaxis in critically ill patients: a systematic review and meta-analysis of randomized trials. Crit Care. 2016;20(1):120.

173. Barletta JF, Bruno JJ, Buckley MS, Cook DJ. Stress Ulcer Prophylaxis. Critical Care Medicine. 2016;44(7):1395-405.

174. Alhazzani W, Alshamsi F, Belley-Cote E, Heels-Ansdell D, Brignardello-Petersen R, Alquraini M, et al. Efficacy and safety of stress ulcer prophylaxis in critically ill patients: a network meta-analysis of randomized trials. Intensive Care Medicine. 2018;44(1):1-11.

175. Deliwala SS, Hamid K, Goyal H, Ponnapalli A, Zayed Y, Bala A, Lakshman H, Malladi S, Jones S, Santana M, Leon B, An MT, Chawla S. Proton Pump Inhibitors Versus Histamine-2-Receptor Antagonists for Stress Ulcer Prophylaxis in Critically Ill Patients: A Meta-analysis and Trial Sequential Analysis. J Clin Gastroenterol. 2022 Mar 1;56(3):204-217.

176. He N, Yan Y, Su S, Ge Q, Zhai S. Are Proton Pump Inhibitors More Effective Than Histamine-2-Receptor Antagonists for Stress Ulcer Prophylaxis in Critically Ill Patients? A Systematic Review and Meta-Analysis of Cohort Studies. Ann Pharmacother. 2022 Sep;56(9):988-997.

177. Lee TC, Goodwin Wilson M, Lawandi A, McDonald EG. Proton Pump Inhibitors Versus Histamine-2 Receptor Antagonists Likely Increase Mortality in Critical Care: An Updated Meta-Analysis. Am J Med. 2021 Mar;134(3):e184-e188.

178. Kow CS, Hasan SS. Use of proton pump inhibitors and risk of adverse clinical outcomes from COVID-19: a meta-analysis. J Intern Med. 2021 Jan;289(1):125-128.

179. Koyyada A. Long-term use of proton pump inhibitors as a risk factor for various adverse manifestations. Therapie. 2021 Jan-Feb;76(1):13-21.

180. Ribiere S, Guillaumot MA, Barré A, Abou Ali E, Barret M, Chaussade S, Coriat R. Quel est le VRAI risque au long cours des inhibiteurs de la pompe à protons ? [What is the REAL long-term risk of proton pump inhibitors?]. Presse Med. 2019 May;48(5):503-510. French. doi: 10.1016/j.lpm.2019.02.011.

181. Liu C, Guo H, Mao H, Tong J, Yang M, Yan X. An Up-To-Date Investigation Into the Correlation Between Proton Pump Inhibitor Use and the Clinical Efficacy of Immune Checkpoint Inhibitors in Advanced Solid Cancers: A Systematic Review and Meta-Analysis. Front Oncol. 2022 Feb 24;12:753234.

182. Rizzo A, Cusmai A, Giovannelli F, Acquafredda S, Rinaldi L, Misino A, Montagna ES, Ungaro V, Lorusso M, Palmiotti G. Impact of Proton Pump Inhibitors and Histamine-2-Receptor Antagonists on Non-Small Cell Lung Cancer Immunotherapy: A Systematic Review and Meta-Analysis. Cancers (Basel). 2022 Mar 9;14(6):1404. doi: 10.3390/cancers14061404.

183. Wei N, Zheng B, Que W, Zhang J, Liu M. The association between proton pump inhibitor use and systemic anti-tumour therapy on survival outcomes in patients with advanced non-small cell lung cancer: A systematic review and meta-analysis. Br J Clin Pharmacol. 2022 Jul;88(7):3052-3063.

184. Perry IE, Sonu I, Scarpignato C, Akiyama J, Hongo M, Vega KJ. Potential proton pump inhibitor-related adverse effects. Ann N Y Acad Sci. 2020 Dec;1481(1):43-58. doi: 10.1111/nyas.14428. Epub 2020 Aug 6. Erratum in: Ann N Y Acad Sci. 2021 Mar;1487(1):74.

185. Rizzo A, Santoni M, Mollica V, Ricci AD, Calabrò C, Cusmai A, Gadaleta-Caldarola G, Palmiotti G, Massari F. The Impact of Concomitant Proton Pump Inhibitors on Immunotherapy Efficacy among Patients with Urothelial Carcinoma: A Meta-Analysis. J Pers Med. 2022 May 20;12(5):842.

186. Veettil SK, Sadoyu S, Bald EM, Chandran VP, Khuu SAT, Pitak P, Lee YY, Nair AB, Antony PT, Ford AC, Chaiyakunapruk N. Association of proton-pump inhibitor use with adverse health outcomes: A systematic umbrella review of meta-analyses of cohort studies and randomised controlled trials. Br J Clin Pharmacol. 2022 Feb;88(4):1551-1566.

Preventive Tx Cardiac/ angioplasty/ concomitant use with dual antiplatelet therapy/ clopidogrel/ aspirin

187. Khan SU, Lone AN, Asad ZUA, Rahman H, Khan MS, Saleem MA, et al. Meta-analysis of efficacy and safety of proton pump inhibitors with dual antiplatelet therapy for coronary artery disease. Cardiovasc Revasc Med. 2019;10:10.

188. Almufleh A, Ramirez FD, So D, Le May M, Chong AY, Torabi N, et al. H2 Receptor Antagonists versus Proton Pump Inhibitors in Patients on Dual Antiplatelet Therapy for Coronary Artery Disease: A Systematic Review. Cardiology. 2018;140(2):115-23.

189. Bundhun PK, Teeluck AR, Bhurtu A, Huang WQ. Is the concomitant use of clopidogrel and Proton Pump Inhibitors still associated with increased adverse cardiovascular outcomes following coronary angioplasty?: a systematic re-

view and meta-analysis of recently published studies (2012 - 2016). BMC Cardiovasc Disord. 2017;17(1):3.

190. Wu W, Liu J, Yu H, Jiang Z. Antiplatelet therapy with or without PPIs for the secondary prevention of cardiovascular diseases in patients at high risk of upper gastrointestinal bleeding: A systematic review and meta-analysis. Exp Ther Med. 2020 Jun;19(6):3595-3603.

191. Dahal K, Sharma SP, Kaur J, Anderson BJ, Singh G. Efficacy and Safety of Proton Pump Inhibitors in the Long-Term Aspirin Users: A Meta-Analysis of Randomized Controlled Trials. American Journal of Therapeutics. 2017;24(5):e559-e69.

192. Demcsak A, Lantos T, Balint ER, Hartmann P, Vincze A, Bajor J, et al. PPIs Are Not Responsible for Elevating Cardiovascular Risk in Patients on Clopidogrel-A Systematic Review and Meta-Analysis. Front Physiol. 2018;9:1550.

193. Hu W, Tong J, Kuang X, Chen W, Liu Z. Influence of proton pump inhibitors on clinical outcomes in coronary heart disease patients receiving aspirin and clopidogrel: A meta-analysis. Medicine (Baltimore). 2018;97(3):e9638.

194. Khan MY, Siddiqui WJ, Alvarez C, Aggarwal S, Hasni SF, Ahmad A, et al. Reduction in postpercutaneous coronary intervention angina in addition to gastrointestinal events in patients on combined proton pump inhibitors and dual antiplatelet therapy: a systematic review and meta-analysis. Eur J Gastroenterol Hepatol. 2018;30(8):847-53.

195. Niu Q, Wang Z, Zhang Y, Wang J, Zhang P, Wang C, et al. Combination Use of Clopidogrel and Proton Pump Inhibitors Increases Major Adverse Cardiovascular Events in Patients With Coronary Artery Disease: A Meta-Analysis. J Cardiovasc Pharmacol Ther. 2017;22(2):142-52.

196. Malhotra K, Katsanos AH, Bilal M, Ishfaq MF, Goyal N, Tsivgoulis G. Cerebrovascular Outcomes With Proton Pump Inhibitors and Thienopyridines: A Systematic Review and Meta-Analysis. Stroke. 2018;49(2):312-8.

197. Li JX, Jin EZ, Li Y, Song ZY, Liu SH, Yan SJ, et al. Lack increased evidence of cardiovascular events in patients receiving clopidogrel with proton-pump inhibitors: A meta-analysis and system review. International Journal of Clinical and Experimental Medicine. 2018;11(11):11481-93.

198. Serbin MA, Guzauskas GF, Veenstra DL. Clopidogrel-Proton Pump Inhibitor Drug-Drug Interaction and Risk of Adverse Clinical Outcomes Among PCI-Treated ACS Patients: A Meta-analysis. J Manag Care Spec Pharm. 2016;22(8):939-47

199. Yi ZM, Qiu TT, Zhang Y, Liu ZY, Zhai SD. Comparison of prophylactic effect of UGIB and effects on platelet function between PPIs and H₂RAs combined with DAPT: systematic review and meta-analysis. Ther Clin Risk Manag. 2017;13:367-77.

200. Wang ZY, Chen M, Zhu LL, Yu LS, Zeng S, Xiang MX, et al. Pharmacokinetic drug interactions with clopidogrel: updated review and risk management in combination therapy. Ther Clin Risk Manag. 2015;11:449-67.

201. Law EH, Badowski M, Hung YT, Weems K, Sanchez A, Lee TA. Association Between Proton Pump Inhibitors and Microscopic Colitis. Annals of Pharmacotherapy. 2017;51(3):253-63

202. Han YY, Li ZX, Duan R. Efficacy and safety of proton pump inhibitors combined with clopidogrel in patients undergoing percutaneous coronary intervention: a meta-analysis. Rev Cardiovasc Med. 2021 Mar 30;22(1):167-174.

203. Guo H, Ye Z, Huang R. Clinical Outcomes of Concomitant Use of Proton Pump Inhibitors and Dual Antiplatelet Therapy: A Systematic Review and Meta-Analysis. Front Pharmacol. 2021 Aug 2;12:694698.

204. Shang YS, Zhong PY, Ma Y, Bai N, Niu Y, Wang ZL. Efficacy and Safety of Proton Pump Inhibitors in Patients With Coronary Artery Diseases Receiving Oral Antiplatelet Agents and/or Anticoagulants: A Systematic Review and Meta-Analysis. J Cardiovasc Pharmacol. 2022 Jul 1;80(1):1-12.

205. Poly TN, Islam MM, Yang HC, Wu CC, Li YJ. Proton pump inhibitors and risk of hip fracture: a meta-analysis of observational studies. Osteoporos Int. 2019;30(1):103-14.

206. Liu J, Li X, Fan L, Yang J, Wang J, Sun J, et al. Proton pump inhibitors therapy and risk of bone diseases: An update meta-analysis. Life Sci. 2019;218:213-23.

207. Lapumnuaypol K, Thongprayoon C, Wijarnpreecha K, Tiu A, Cheungpasitporn W. Risk of Fall in Patients Taking Proton Pump Inhibitors: A Meta-Analysis. QJM : monthly journal of the Association of Physicians. 2019;112(2):115-21.

208. Fan XD, Ayom MIN, Sun WG, Yin PP, Wang XY, Jia A, et al. An updated meta-analysis: The effect of proton pump inhibitor on risk of osteoporosis and fracture. International Journal of Clinical and Experimental Medicine. 2017;10(11):15680-95.

209. Dubcenco E, Beers-Block PM, Kim LP, Schotland P, Levine JG, McCloskey CA, et al. A Proton Pump Inhibitor in the Reformulation Setting: Bioequivalence and Potential Implications for Long-Term Safety. Clinical and Transla-

tional Science. 2017;10(5):387-94.

210. Hussain S, Siddiqui AN, Habib A, Hussain MS, Najmi AK. Proton pump inhibitors' use and risk of hip fracture: a systematic review and meta-analysis. Rheumatology international. 2018;38(11):1999-2014.

211. Nassar Y, Richter S. Proton-pump Inhibitor Use and Fracture Risk: An Updated Systematic Review and Meta-analysis. J. 2018;25(3):141-51.

212. Yang SD, Chen Q, Wei HK, Zhang F, Yang DL, Shen Y, et al. Bone fracture and the interaction between bisphosphonates and proton pump inhibitors: a meta-analysis. International journal of clinical and experimental medicine. 2015;8(4):4899-910.

213. Cai D, Feng W, Jiang Q. Acid-suppressive medications and risk of fracture: An updated meta-analysis. International Journal of Clinical and Experimental Medicine. 2015;8(6):8893-904.

214. Aleraij S, Alhowti S, Ferwana M, Abdulmajeed I. Effect of proton pump inhibitors on bone mineral density: A systematic review and meta-analysis of observational studies. Bone Rep. 2020 Nov 10;13:100732.

215. Briganti SI, Naciu AM, Tabacco G, Cesareo R, Napoli N, Trimboli P, Castellana M, Manfrini S, Palermo A. Proton Pump Inhibitors and Fractures in Adults: A Critical Appraisal and Review of the Literature. Int J Endocrinol. 2021 Jan 15;2021:8902367.

216. Chen Jiayun, Li Anan, Lü Zhaohui, Wu Zixuan, Cai Minjie, Huang Xuyan . Effect of long-term use of proton pump inhibitors on bone mineral density and bone metabolism: a Meta-analysis[J]. Chinese Journal of Tissue Engineering Research, 2021, 25(17): 2775-2780.

217. Li J, Xie X, Liu W, Gu F, Zhang K, Su Z, Wen Q, Sui Z, Zhou P, Yu T. Acid-Suppressive Drugs and Risk of Fracture in Children and Young Adults: A Meta-Analysis of Observational Studies. Front Pharmacol. 2021 Aug 6;12:712939.

METHODS

Search Strategy

The information specialist searched the following databases from 2013 until the end of June 2022 without restrictions to publication status or language: Ovid MEDLINE, Ovid Embase, and Epistemonikos. The Cochrane Central Register of Controlled Trials (CENTRAL) was searched without date limits. We present below the full MEDLINE search strategy from which the additional database strategies were derived:

Database: Ovid MEDLINE(R) ALL <1946 to June 29, 2022>

Search Date: 30 June 2022

----

1 exp Proton Pump Inhibitors/ (20651)

2 exp 2-Pyridinylmethylsulfinylbenzimidazoles/ (11708)

3 (proton adj2 pump adj2 antagon*).tw,kf. (333)

4 (proton adj2 pump adj2 block*).tw,kf. (232)

5 (proton adj2 pump adj2 inhibitor*).tw,kf. (16683)

6 (PPI or PPIs).tw,kf. (26512)

7 (hydrogen potassium adj3 antagon*).tw,kf. (2)

8 (hydrogen potassium adj3 block*).tw,kf. (1)

9 (hydrogen potassium adj3 inhibit*).tw,kf. (15)

10 Dexlansoprazole/ (78)

11 (dexlansoprazole or dexilant or kapidex).tw,kf. (136)

12 Esomeprazole Sodium/ (1177)

13 (esomeprazole or alenia or escz or esofag or esomeprazol or esoprax or esotrex or inexium or nexiam or nexlum or

perprazole or sompraz).tw,kf. (1789)

14 (ilaprazole or noltec).mp. (80)

15 Lansoprazole/ (2176)

16 (lansoprazole or agopton or bamalite or banilux or betalans or compraz or dakar or daxar or dostab or duomate or ilsatec or inhipraz or inhibitol or keval or lancid or lacopen or langaton or lanpra or lanpraz or lanprol or lanproton or lansazol or lansobene or lansol or lansone or lansop or lansopep or lansoprazol or lansox or lansozole or lanster or lanston or lanvell or lanximed or lanzo or lanzol-30 or lanzopral or lanzoprazole or lanzor or lanzul or lapraz or laproton or lasgan or levant or limpidex or lopral or lupizole or monolitum or ogast or ogasto or ogastoro or ogastro or opiren or pamper or praton or prevacid or prezal or prolanz or prosogan or pysolan or promeco or pro ulco or sopralan or suprecid or takepron or tanzolan or ulpax or zoton).tw,kf. (5473)

17 Omeprazole/ (8913)

18 (omeprazole or aleprozil or antra or arapride or audazol or baromezole or desec or dointol or domer or dudencer or duogas or emeproton or epirazol or ezipol or gasec or gastec or gastop or gastrimut or gastrolac or gastroloc or glaveral or hovizol or hyposec or inhibitron or inhipump or logastric or lomac or lopraz or losamel or losec or losecosan or ludea or madiprazole or maxor or medoprazole or medral or meiceral or mepral or mepzol or mezzopram or miol or miracid or mopral or mopralpro or nexium or nocid or ocid or ogal or olex or olexin or omedar or omelon or omep uno or omepral or omeprazen or omeprazol or omeprazon or omepril or omeraz or omesec or omestad or omez or omezin or omezol or omezolan omezole or omezzol or omisec or omizac or omolin or ompranyt or omprazole or onexal or oprax or ozoken or parizac or penrazole or pepticum or peptidin or peptilcer or peptizole or pra-sec or prazidec or prazole or prilosec or probitor or proceptin or protoloc or ramezol or rapinex or reglacid or risek or romep or roweprazol or secrepina or severon or stomacer or stomec or stozole or suifac ulcerol or ulcozol or ulnor or ulsek or ulsen or ulzol or vulcasid or wonmp or xoprin or zatrol or zefxon or zegerid or zenpro or zimor or zoltum).tw,kf. (9365)

19 Pantoprazole/ (1415)

20 (pantoprazole or anagastra or branzol or controloc or eupantol or inipom or inipomp or pantecta or pantodac or pantodar or pantoloc or pantop or pantozol or pepticus or protium or protonix or rifun or somac or ulcepraz or ulcotenal or zentro or ziprol or zurcal or zurcale or zurcazol).tw,kf. (2245)

21 Rabeprazole/ (1027)

22 (rabeprazole or aciphex or dexrabeprazole or pariet or pariprazole or rabec or rabeloc).tw,kf. (1293)

23 or/1-22 (53370)

24 (ae or co or de).fs. (6637288)

25 (safe or safety or side effect or side effects or unsafe).ti,kf. (238114)

26 (safe or safety or side effect or side effects or unsafe).ab. /freq=2 (320082)

27 ((adverse or fatal or harm* or serious or toxic or undesirable) adj3 (effect* or event* or outcome* or patient* or reaction*)).ab,kf. (660046)

28 (adrs or complication or complicationoxious or teratogen* or tolerable or tolerability or toxicity or treatment emergent).ti,kf. (186384)

29 (adrs or complication or complications or noxious or teratogen* or tolerable or tolerability or toxicity or treatment emergent).ab. /freq=2 (492078)

30 (cessat* or discontinu* or reduce or reduced or reduces or reducing or reduct* or stop* or taper* or withdraw*).tw,kf. (4250194)

31 (death or deaths or fatal or fatalities or fatality or warning).ti. (182962)

32 exp product surveillance, postmarketing/ (17584)

33 exp adverse drug reaction reporting systems/ (8512)

34 exp clinical trials, phase iv/ (363)

35 exp *drug hypersensitivity/ (36981)

36 exp *drug toxicity/ (90543)

37 exp "drug-related side effects and adverse reactions"/ (127663)

38 substance-related disorders/ (102665)

39 abnormalities, drug-induced/ (14713)

40 exp brain diseases/ (1351684)

41 exp confusion/ (16343)

42 exp neurocognitive disorders/ (294526)

43 (alzheimer* or amnesi* or confus* or deliri* or dementia).mp. (374982)

44 (cognit* adj2 (declin* or impair*)).mp. (114157)

45 exp fractures, bone/ (201172)

46 (bone* adj2 (broken or fractur*)).mp. (81936)

47 bone density/ (58811)

48 (bone densit* or bone mineral content* or bone mineral densit*).mp. (91273)

49 (bone* adj2 strength*).mp. (7594)

50 magnesium deficiency/ (4408)

51 (magnesium adj2 deficien*).mp. (5116)

52 (hypomagnesemia* or hypomagnesaemia*).mp. (4375)

53 exp vitamin b 12 deficiency/ (11701)

54 (vitamin adj (b12 or b 12) adj2 deficien*).tw,kf. (4816)

55 anemia, iron deficiency/ (11247)

56 (anaemi* or anemi* or iron deficien*).mp. (222634)

57 exp pneumonia/ (268764)

58 (lung inflammat* or pneumonia* or pneumonitides or pneumonitis or pulmonary inflammat*).mp. (298999)

59 acid rebound.tw,kf. (52)

60 exp acute kidney injury/ (53328)

61 (acute kidney failure* or acute kidney injur* or acute kidney insufficienc* or acute renal failure* or acute renal injur* or acute kidney insufficienc*).mp. (76253)

62 exp neoplasms/ (3705208)

63 (carcinoid* or cancer* or malignan* or neoplasm* or polyp* or precancer* or premalignan*).mp. (4203335)

64 exp enterobacteriaceae infections/ (108170)

65 ((cronobact* or enteric or enterobacteri*) adj2 infection*).mp. (14443)

66 exp clostridium Infections/ (32001)

67 ((clostridium or c difficile) adj2 infection*).mp. (14213)

68 diarrhea/ (51110)

69 (diarrhea* or diarrhoea*).mp. (135178)

70 (angina* or cardiac or cardiovascular or coronary or heart or infarct* or myocardi* or preinfarct*).mp. (2519554)

71 gastriti*.mp. (32067)

72 metaplasia*.mp. (22467)

73 dysplasi*.mp. (89884)

74 or/24-73 (16049792)

75 systematic review/ (200284)

76 meta analysis.mp,pt. (247557)

77 ((systematic adj3 review*) or (systematic adj2 overview*)).tw,kf. (263925)

78 (embase or medline or pubmed or searched).ab. (340995)

79 or/75-78 (533776)

80 23 and 74 and 79 (1624)

81 animals/ not (humans/ and animals/) (4989046)

82 80 not 81 (1608)

83 82 and (2013* or 2014* or 2015* or 2016* or 2017* or 2018* or 2019* or 2020* or 2021* or 2022*).ed,dt. (1120)

INCLUSION CRITERIA

Systematic reviews (with or without meta-analysis) were included that met the following criteria: (Cochrane "PICOS" format):

P—adults aged 18 years or older.

I—PPI therapy for any indication for duration of more than 12 weeks.

C—Non-use or histamine type-2 receptor antagonist (H2RA) use.

O—any adverse events
